# Induced somatic mutation accumulation during skeletal muscle regeneration reduces muscle strength

**DOI:** 10.1038/s43587-025-00941-y

**Published:** 2025-08-20

**Authors:** Peter Vrtačnik, Lara G. Merino, Santhilal Subhash, Hafdís T. Helgadóttir, Matthieu Bardin, Fabiana Stefani, Depin Wang, Ping Chen, Irene Franco, Gwladys Revêchon, Maria Eriksson

**Affiliations:** 1 Department of Medicine, Huddinge, Karolinska Institutet, Huddinge, Sweden; 2 Department of Biosciences and Bioengineering, Indian Institute of Technology Jammu, Jammu, India; 3 Research Program of Stem Cells and Metabolism, Faculty of Medicine, University of Helsinki, Helsinki, Finland; 4 Division of Clinical Chemistry, Department of Laboratory Medicine, Karolinska Institutet, Stockholm, Sweden; 5 Division of Genetics and Cell Biology, IRCCS San Raffaele Scientific Institute, Milan, Italy; 6 Division of Genetics and Cell Biology, San Raffaele University, Milan, Italy

**Keywords:** Diseases, Ageing, Experimental models of disease, DNA mismatch repair, Genomic instability

## Abstract

Aging is associated with a progressive decline in tissue function and regenerative capacity, partly due to genomic instability, one of the hallmarks of aging^1,2^. Genomic instability encompasses DNA damage and the accumulation of somatic mutations in post-zygotic cells, yet the specific impact of these mutations on age-related tissue dysfunction remains poorly understood. To address this, we developed a mouse model in which genomic instability was induced specifically in muscle progenitor cells^3^ through targeted deletion of the *Msh2* (ref. ^4^) and *Blm*^5^ genes. This allowed us to assess how elevated DNA damage and somatic mutations, from single-nucleotide variants (SNVs) to structural variants, affect muscle regeneration following injury. These mice exhibited impaired muscle regeneration, characterized by smaller muscle fibers, reduced muscle mass gain and decreased grip strength. Importantly, similar muscle deficits were observed in a second mouse model where somatic mutations were elevated with less substantial DNA damage. These findings provide evidence that the accumulation of somatic mutations can potentially compromise the function of somatic cells, contributing to the aging phenotype in skeletal muscle.

## Abstract (web summary)

With aging, somatic mutations accumulate in cellular DNA; however, whether they drive age-related functional decline is incompletely understood. Here the authors show that these mutations can weaken muscle repair and reduce strength after injury, suggesting they play a role in age-related physical decline in mouse muscle.

## Main

Old age is a major risk factor for diseases such as cancer, type 2 diabetes, cardiovascular disease and neurodegeneration. Aging is linked to reduced tissue function and regeneration^1^, with genomic instability, marked by accumulating somatic mutation, being a key hallmark^1,2^. These mutations, arising from replication errors or DNA repair defects, are not inherited but lead to tissue mosaicism^2^. Although genome instability and DNA damage have been characterized in aging^6–9^, the functional role of somatic mutation accumulation in age-related tissue decline and age-related diseases beyond cancer remains less explored^10,11^.

Whole-genome sequencing (WGS) studies have shown that somatic mutations accumulate with age in human skeletal muscle progenitor cells^12^ and other tissues^13–16^, with similar observations in most tumor types^17,18^. Differentiated cells often carry even higher mutation loads^13,19^, highlighting the underestimated extent of age-related somatic mutagenesis. Although we previously showed that high mutation burden impairs satellite cell (SC) function in vitro^12^, in vivo evidence for the role of somatic mutations in muscle tissue function remains limited.

Aged human cells, including SCs, show structural genetic variations such as chromosomal aberrations, SNVs and short insertions/deletions (InDels)^12,13,15^. To model this, we generated muscle somatic mutator (MSM) mice by deleting the DNA repair genes *Msh2* and *Blm* specifically in SCs. *Msh2* deficiency, linked to Lynch syndrome, leads to increased SNVs, InDels, and chromatin breaks^4,20–23^, though patients do not show muscle impairment^24^. Locomotor defects have only been shown in *Msh2*-deficient mice in correlation with altered neuron firing^25^. *Blm* mutations, which cause Bloom syndrome, increase chromosomal instability and structural genetic variations without affecting muscle development^5,21,26,27^. To study the effects of increased somatic mutations in SCs, we deleted *Msh2* and *Blm* and then induced SC-driven muscle regeneration following injury. Expanded recombined SCs led to impaired muscle regeneration and reduced strength. To try to separate the effect of somatic mutations from those of DNA damage, we created a second model with *Msh2* deletion alone, which induced SNVs and InDels with less substantial DNA damage. This model replicated the MSM phenotype, supporting a somatic mutation driven effect on muscle regeneration. In summary, our strategy offers a framework to study the functional impact of somatic mutations in diverse cell types and their potential role in tissue dysfunction.

## Generation of the MSM model

**Fig. 1:**
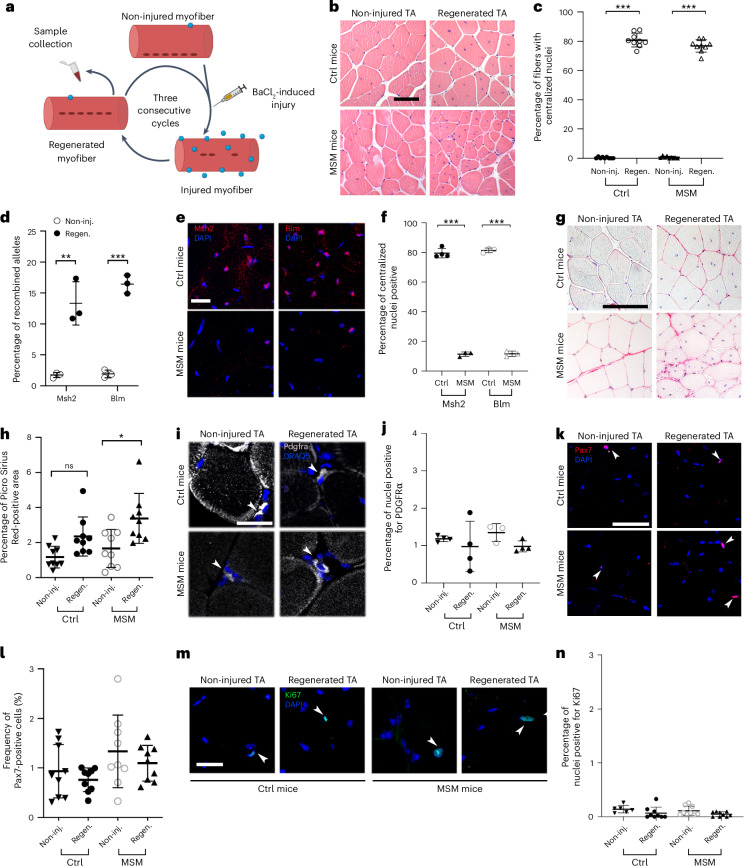
Characterization of regeneration in control and MSM mice. **a**, Schematic representation of the experimental setup. Created in BioRender. Eriksson. M (2025). https://BioRender.com/qb882vw. **b**, Hematoxylin-eosin TA staining after three regeneration cycles. The right TA is regenerated muscle, and the uninjured left TA is the control (Ctrl). **c**, Quantification of fibers with centralized nuclei in non-injured and regenerated TA (control *n* = 9, MSM *n* = 9); Ctrl-non-inj. vs Ctrl-Regen. *P* = 1 ×10^−13^; MSM-non-inj. vs MSM-Regen. *P* = 1 ×10^−13^. **d**, *Msh2* and *Blm* allele recombination quantification in non-injured and regenerated TA of MSM mice (non-injured *n* = 3, regenerated *n* = 3); *Msh2* recombination: non-inj. *vs* Regen. *P* = 0.0047; *Blm* recombination: non-inj. vs Regen *P* = 9.89 ×10^−5^. **e**, Msh2 and Blm immunofluorescence of non-injured and regenerated TA. **f**, Quantification of Msh2-positive nuclei in non-injured and regenerated TA (control *n* = 4, MSM *n* = 3) and Blm-positive nuclei in non-injured and regenerated TA (Control *n* = 3, MSM *n* = 3); Msh2 expression: Ctrl *vs* MSM *P* = 8.07×10^−10^; Blm expression: Ctrl *vs* MSM *P* = 9.13 × 10^−^^10^. **g**, Picro Sirius Red staining of non-injured and regenerated TA. **h**, Quantification of Picro Sirius Red-stained muscle in non-injured and regenerated TA (Control *n* = 9, MSM *n* = 9); Ctrl-Non-inj. *vs* Ctrl-Regen. *P* = 0.1269; MSM-non-inj. *vs* MSM-Regen. *P* = 0.0120. **i**, Pdgfrα immunofluorescence of non-injured and regenerated TA. **j**, Quantification of Pdgfrα-positive cells in non-injured and regenerated TA of control mice (Non-injured TA *n* = 4, regenerated TA *n* = 4) and MSM mice (Non-injured TA *n* = 3, regenerated TA *n* = 4) Ctrl-Non-inj. *vs* Ctrl-Regen. *P* = 0.8625; MSM-non-inj. *vs* MSM-Regen. *P* = 0.5965. **k**, Pax7 immunofluorescence of non-injured and regenerated TA. **l**, Quantification of Pax7-positive nuclei in non-injured and regenerated TA (Control *n* = 9, MSM *n* = 9); Ctrl-Non-inj. *vs* Ctrl-Regen. *P* = 0.8868; MSM-non-inj. *vs* MSM-Regen. *P* = 0.7465. **m**, Ki67 immunofluorescence of non-injured and regenerated TA. **n**, Quantification of Ki67 positive nuclei (Control-non-inj *n* = 6, Control-regen *n* = 9, MSM-non-inj *n* = 9, MSM-regen *n* = 9); Ctrl-Non-inj. *vs* Ctrl-Regen. *P* = 0.3449; MSM-non-inj. *vs* MSM-Regen. *P* = 0.3900. Panels **b** and **g** scale bars indicate 100 μm; panels **e**, **i**, **k** and **m** scale bars indicate 20 μm. Statistical tests used in **c**, **f**, **h**, **j**, **l** and **n** were one-way analysis of variance (ANOVA) with Tukey multiple comparison test (**d**, unpaired *t*-test, two tailed). * *P* < 0.05; ** *P* < 0.01; *** *P* < 0.001. Graphs in **c**, **d**, **f**, **h**, **j**, **l** and **n** present data as mean ± standard deviation (s.d.). Arrowheads in **i**, **k** and **m** indicate positive nuclei/cells. Source data

We created the MSM mouse model by combining a *Pax7*-driven tamoxifen-inducible Cre recombinase^3^ with floxed *Msh2*^4^ and *Blm*^5^ alleles. Recombination was induced postnatally (weeks 7–9) specifically in SCs, which drive muscle regeneration^5,28,29^. To enhance mutagenesis and assess SC function in response to increased DNA damage and somatic mutations, we subjected each mice to three consecutive BaCl_2_ induced injury-regeneration cycles in the right tibialis anterior (TA) muscle (Fig. 1a). The contralateral left TA functioned as uninjured muscle control. Mice carrying only the Pax7-driven Cre recombinase and not deficient for *Msh2* and *Blm*, were also subjected to injury-regeneration cycles in the right TA (referred to as control mice). TAs were analyzed after the third cycle.

## Successful *Msh2* and *Blm* recombination in MSM skeletal muscle

To assess regeneration, we quantified the number of fibers with centralized nuclei in the right TA, a hallmark of regenerated muscle (Fig. 1b). Control and MSM mice showed, on average, 80.8% and 76.9% of fibers with centralized nuclei, respectively, whereas the uninjured left TA contained virtually none, confirming right TA regeneration after injury (Fig. 1c). To evaluate *Msh2* and *Blm* deletion, we measured allele recombination in non-injured and regenerated muscle. Although the average percentage of recombined alleles was 1.72% for *Msh2* and 1.92% for *Blm* in non-injured TA, it increased to 13.33% for *Msh2* and 16.43% for *Blm* in regenerated MSM muscle (Fig. 1d), indicating successful SC targeting and expansion. Immunofluorescence confirmed this, with *Msh2* and *Blm* signals dropping from 79.6% and 81.5% stained centralized nuclei in control mice regenerated muscle, to 11.4% and 11.6% stained centralized nuclei in MSM mice regenerated muscle (Fig. 1e,f).

## Reduced TA regeneration efficiency in MSM mice after injury

We examined muscle regeneration in control and MSM mice. In controls, connective tissue, typically elevated during regeneration^3^, returned to baseline in regenerated TA. By contrast, MSM mice showed persistently elevated collagen in regenerated TA compared to non-injured TA, indicating impaired repair (Fig. 1g,h). To determine if this was due to sustained fibroadipogenic progenitor (FAP)^30,31^ expansion, we performed Pdgfrα staining^30,31^. FAP numbers were similar in non-injured and regenerated TA across both groups (Fig. 1i,j), suggesting fibrosis in MSM mice is not due to chronic FAP accumulation. After the final regeneration cycle, Pax7 staining showed similar SC frequencies in MSM and control mice (0.8 to 1.3%, on average), consistent with basal quiescent levels (~1%)^32^ in both non-injured and regenerated TA (Fig. 1k,l). Ki67 staining also revealed no difference in proliferating cells between groups (Fig. 1m,n), suggesting no changes in basal SCs proliferative status and that regeneration had ceased.

## Increased mutations and genomic instability in MSM muscle

**Fig. 2:**
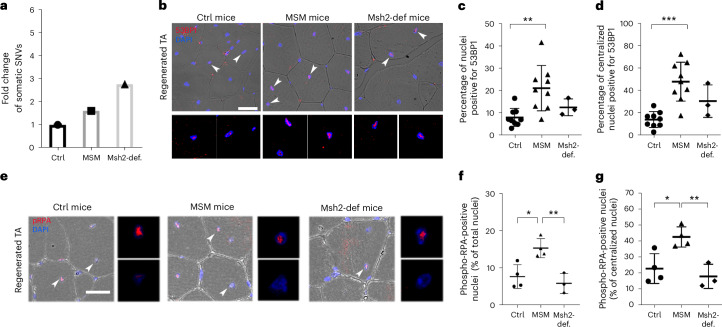
Increased number of detected somatic mutations and DNA DSBs in regenerated skeletal muscle of MSM/Msh2-def mice. **a**, Barplot of the number of somatic single-nucleotide variants (SNVs) in by WGS of representative histological samples of regenerated TA from control, MSM and *Msh2*-deficient mice. The measurement was performed on downsampled data (*n* = 3). **b**, 53BP1 immunofluorescence of regenerated TA. Arrows indicate 53BP1-positive nuclei that are enlarged in the below pictures. Transmitted detection was used to visualize myogenic fibers. **c**, Quantification of 53BP1-positive cells in regenerated TA (Control *n* = 9, MSM *n* = 9, Msh2-def. *n* = 3); Control vs MSM *P* = 0.0039, Control vs Msh2-def. *P* = 0.6368. **d**, Quantification of 53BP1-positive centralized myonuclei in regenerated TA (Control *n* = 9, MSM *n* = 9, Msh2-def. *n* = 3); Control vs MSM *P* = 0.0001, Control vs Msh2-def. *P* = 0.1845. **e**, Phospho-RPA32 (phospho S4 + S8) immunofluorescence of regenerated TA. Arrowheads indicate one phospho-RPA32 (phospho S4 + S8)-positive and one negative nuclei that are enlarged in the below pictures. Transmitted detection was used to visualize myogenic fibers. **f**, Quantification of phospho-RPA32 (phospho S4 + S8)-positive nuclei in regenerated TA (Control *n* = 4, MSM *n* = 4, Msh2-def. *n* = 3); Control vs MSM *P* = 0.0131, MSM *vs* Msh2-def. *P* = 0.0061. **g**, Quantification of phospho-RPA32 (phospho S4 + S8)-positive centralized myonuclei in regenerated TA (Control *n* = 4, MSM *n* = 4, Msh2-def. *n* = 3); Control vs MSM *P* = 0.0187, MSM vs Msh2-def. *P* = 0.0087. Panel **b** scale bar indicates 100 μm; panel **e** scale bar indicates 20 μm. Statistical tests used in **c**, **d**, **f** and **g**: one-way ANOVA with Tukey multiple comparison test. * *P* < 0.05; ** *P* < 0.01; *** *P* < 0.001. Graph **a** presents a single representative value for each group. Graphs in **c**, d, **f** and **g** present data as mean ± s.d. Source data

To confirm increased somatic mutations in the MSM model, we performed deep WGS of regenerated muscle. MSM mice showed a 1.61-fold increase in the number of SNVs compared to control mice (Fig. 2a). Structural variant analysis also revealed more large deletions in MSM compared to control mice muscle, consistent with *Blm* deletion^27^ (Extended Data Fig. 2a,b and Supplementary Data 1). 53BP1 immunofluorescence showed elevated double-strand breaks (DSBs) in MSM-regenerated muscle (Fig. 2b,c), supporting increased DNA damage^33^. Additionally, regenerated MSM muscles had more for phosphorylated RPA-positive (S4/S8) cells, a marker of single-stranded DNA, present during DSB repair^34^ (Fig. 2e,f). Both markers were enriched in centralized nuclei of MSM muscle fibers (Fig. 2d,g).

## Accumulation of somatic mutations in Msh2-deficient muscle

**Extended Data Fig. 1:**
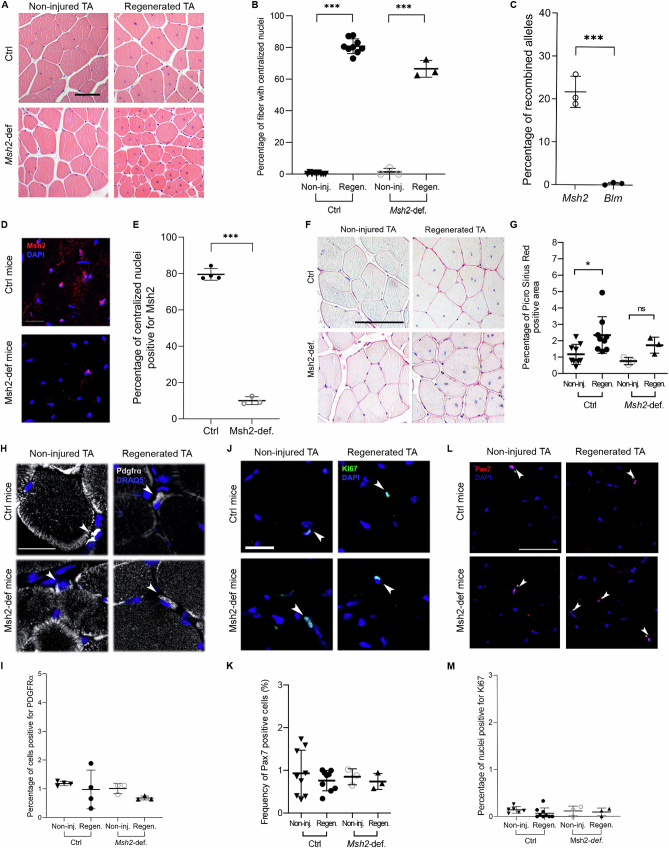
Characterization of regeneration in *Msh2*-deficient TA. (**a**) Hematoxylin-eosin staining of non-injured and regenerated TA after 3 cycles of injury and regeneration. (**b**) Quantification of fibers with centralized nuclei in non-injured and regenerated TA (Control n = 9, *Msh2*-def. n = 3); Ctrl-non-inj. *vs* Ctrl-Regen. P = 2.3e-14; *Msh2*-def. non-inj. *vs* *Msh2*-def. Regen. P = 2.8e-14. (**c**) Quantification of *Msh2*/*Blm* recombination in regenerated TA after 3 regeneration cycles (n = 3; P = 0.0005). (**d**) Msh2 immunofluorescence of regenerated TA. (**e**) Quantification of Msh2-positive centralized nuclei in regenerated TA (Control n = 4, *Msh2*-def. n = 3); Control vs *Msh2*-def P = 5.8e-07. (**f**) Picro Sirius Red staining of non-injured and regenerated TA after 3 regeneration cycles. (**g**) Quantification of Picro Sirius Red-stained muscle in non-injured and regenerated TA (Control n = 9, *Msh2*-def. n = 3); Ctrl-Non-inj. *vs* Ctrl-Regen. P = 0.0311; *Msh2*-def. non-inj. *vs* *Msh2*-def. Regen. P = 0.4906. (**h**) Pdgfrα immunofluorescence of non-injured and regenerated TA. (**i**) Quantification of Pdgfrα-positive cells in non-injured and regenerated TA after 3 cycles of regeneration (Control n = 4, *Msh2*-def. n = 3); Ctrl-Non-inj. *vs* Ctrl-Regen. P = 0.8669; *Msh2*-def. non-inj. *vs* *Msh2*-def. Regen. P = 0.7195. (**j**) Pax7 immunofluorescence of non-injured and regenerated TA. (**k**) Quantification of Pax7-positive cells in non-injured and regenerated TA after 3 regeneration cycles (Control n = 9, *Msh2*-def. n = 3); Ctrl-Non-inj. *vs* Ctrl-Regen. P = 0.7764; *Msh2*-def. non-inj. *vs* *Msh2*-def. Regen. P = 0.9846. (**l**) Ki67 immunofluorescence of non-injured and regenerated TA. (**m**) Quantification of Ki67 positive cells in non-injured and regenerated TA after 3 cycles of injury and regeneration (Control-non-inj n = 6, Control-regen n = 9, *Msh2*-def-non-inj n = 3, *Msh2*-def-regen n = 3) Ctrl-Non-inj. *vs* Ctrl-Regen. P = 0.4816; Msh2-def. non-inj. *vs* Msh2-def. Regen. P = 0.9924. Panels **a** and **f** scale bars indicate 100 μm; panels **d**, **h**, **j** and **l** scale bars indicate 20 μm. Statistical test: **c** and **e**: unpaired *t*-test two tailed; **b**, **g**, **i**, **k** and **m**: one-way ANOVA with Tukey multiple comparison test. Graphs **b**, **c**, **e**, **g**, **i**, **k**, and **m** present data as mean ± SD. Note: for panels **a**, **b**, **d**–**m** the control images and control quantifications correspond to those in Fig. 1b, c, e–m. **h**, **j**, **l** Arrows indicate positive nuclei/cells. Source data

To try to separate persistent DNA damage effects from those of somatic mutations, we generated a second mouse model with *Pax7*-specific deletion of *Msh2* (hereafter “*Msh2*-deficient”), which leads to SNV and InDel accumulation but not structural variants^3,4^. After three injury-regeneration cycles, *Msh2*-deficient mice muscles resembled MSM histologically, with 60.7% of fibers containing central nuclei (Extended Data Fig. 1a,b). 21.7% of *Msh2* alleles were recombined (Extended Data Fig. 1c), and only 10% of centralized nuclei were Msh2-positive, compared to 79.6% in control mice regenerated muscle (Extended Data Fig. 1d,e).

**Extended Data Fig. 2:**
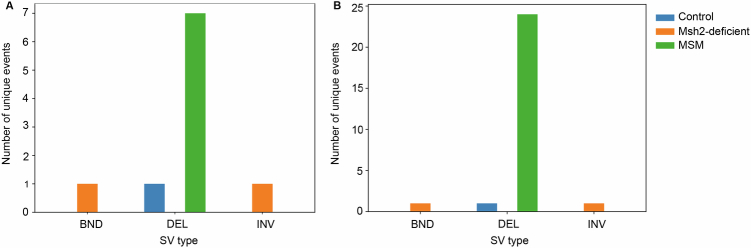
MSM mice accumulate more SVs than control and *Msh2*-deficient mice. Quantification of somatic structural variants (SVs) in skeletal muscle cells by WGS of representative histological samples of regenerated TA from control, MSM and *Msh2*-deficient mice. Analysis was performed both on downsampled data (**a**) and non-downsampled data (**b**). BND = interchromosomal translocation, DEL = deletion, INV = inversion. Source data

*Msh2*-deficient mice showed a 2.77-fold increase in SNVs compared to controls (Fig. 2a), with no increase in SVs (Extended Data Fig. 2a,b and Supplementary Data 1). Consistent with this, there was no statistically significant difference in the frequency of 53BP1-positive cells, suggesting that the elevated DSBs in MSM muscle might be mostly linked to *Blm* deletion (Fig. 2b–d). Phosphorylated RPA levels also remained unchanged (Fig. 2e–g), indicating minimal activation of mismatch repair (MMR), nucleotide excision repair or base excision repair^34^.

*Msh2*-deficient mice showed no changes in connective tissue or FAP frequency after the last regeneration cycle, similar to controls and unlike MSM mice (Extended Data Fig. 1f–i). The number of Pax7-positive cells returned to baseline, with no active proliferation detected (Extended Data Fig. 1j–m).

## Somatic SNV-associated genes related to muscle regeneration

**Fig. 3:**
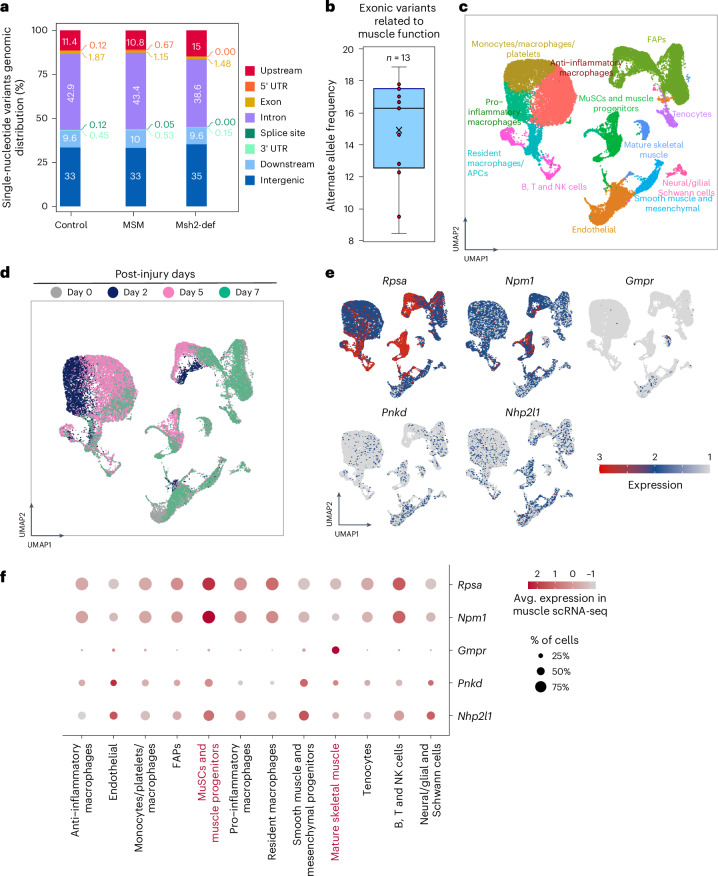
Somatic variants and corresponding genes related to muscle regeneration. **a**, Graphic representation of the genomic distribution of somatic variants identified from non-downsampled control, Msh2-deficient and MSM mice. **b**, Distribution of alternate allele frequency of 13 somatic variants on exons of protein coding gene. The central line of the boxplot represents the mean value of the data. The edges of the box correspond to the 25^th^ (Q1) and 75^th^ percentile (Q3). The whiskers extend to the most extreme data points within 1.5 times the interquartile range from the lower and upper quartiles, and points beyond whiskers are outliers. **c**, Cell types derived by reanalyzing single-cell transcriptome dataset of regenerating muscle of an injured mice from the study published by De Micheli et al.^42^ **d**, Distribution of cells from non-injured (Day 0) and days post-injury (Day 2, Day 5 and Day 7) in individual clusters obtained from single-cell transcriptome analysis. **e**, Expression of protein coding genes associated with somatic variants visualized using UMAP (uniform manifold approximation and projection). **f**, Expression of protein coding genes associated with somatic variants from control, Msh2-deficient and MSM myogenic cells. The circle size represents percentage of cells in which corresponding gene is expressed and the color gradient represents average expression level in individual clusters or corresponding cell types. NK, natural killer. Source data

**Extended Data Fig. 3:**
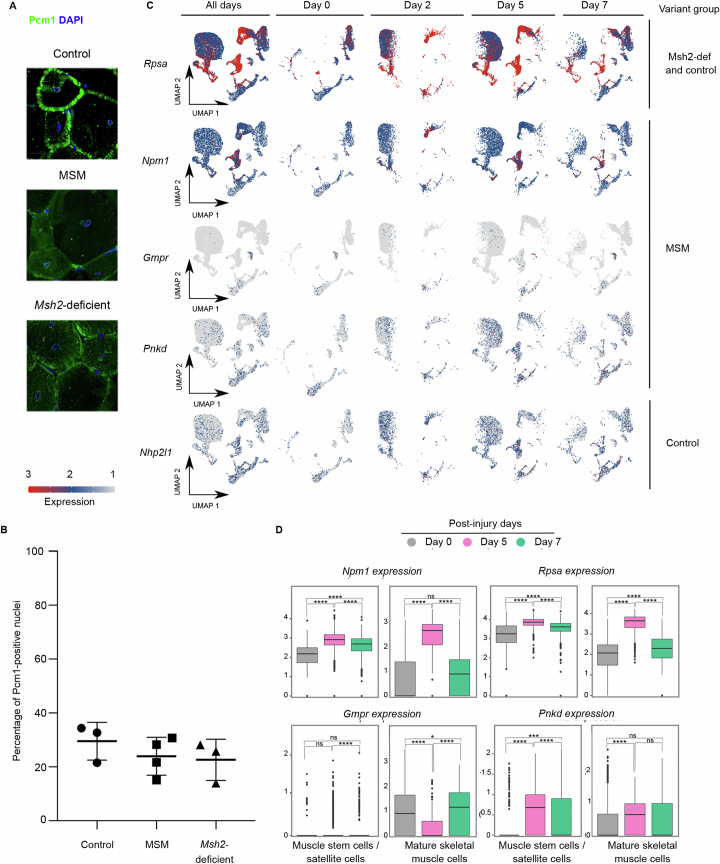
Somatic variants and corresponding genes related to muscle regeneration. (**a**) Pcm1 immunofluorescence of regenerated TA. (**b**) Quantification of Pcm1-positive nuclei in regenerated TA (Control n = 3, MSM n = 4, *Msh2*-def. n = 3); Control vs MSM P = 0.5898, Control vs *Msh2*-def. P = 0.5022. (**c**) Expression of protein coding genes associated with somatic variants visualized in uninjured (Day 0) and post-days of injury (Day 2, 5, and 7) using UMAP (Uniform Manifold Approximation and Projection). (**d**) Boxplots with expression levels of protein coding genes associated with exonic somatic variants in Day 0, Day 5, and Day 7 in muscle associated cell types. The central line of the boxplots represents the mean value of the data. The edges of the boxes correspond to the 25^th^ (Q1) and 75^th^ percentile (Q3). The whiskers extend to the most extreme data points within 1.5 times the interquartile range from the lower and upper quartiles, and points beyond whiskers are outliers. P-values were determined using the Wilcoxon test and are denoted by *<0.05, **<0.01, ***<0.001, **** <0.0001, ns is non-significant and the p-values are adjusted for multiple testing correction by Benjamini Hochberg method Panel **a** scale bar indicates 20 μm. Panel **b** was analyzed with one-way ANOVA with Tukey multiple comparison test. Graph **b** presents data as mean ± SD. Source data

WGS analysis detected 828, 692 and 2,127 somatic variants in regenerated TA from control, *Msh2*-deficient, and MSM mice, respectively (Supplementary Data 2), with similar genomic distributions across groups (Fig. 3a). We found 35 exonic SNVs affecting the protein sequence (Supplementary Table 1). We used MutPred2^35^ on the 20 missense SNVs, and 5 were predicted as likely deleterious (Supplementary Table 1). Thirteen of the 35 exonic mutations occurred in genes related to muscle biology, including *Rpsa*, *Gmpr*, *Npm1*, *Pnkd* and *Nhp2l1* (refs. ^36–40^). The alternative allele frequencies of these SNVs ranged from 8.5% to 19%, indicating their presence in a substantial fraction of cells (Supplementary Table 1 and Fig. 3b). Pcm1 staining^41^ showed that about 25.4% of nuclei (range: 13–32%) belonged to muscle fibers (Extended Data Fig. 3a,b), suggesting these mutations may be present in newly formed fibers, although their presence in other cell types proliferating during regeneration cannot be excluded^36,42^.

To explore links between somatic variants and an impaired muscle phenotype, we analyzed the function of the mutated genes using a published scRNA-seq dataset of muscle regeneration following injury^42^. Cell clusters from injured muscle (up to Day 7 post-injury) were identified (Fig. 3c,d), and gene expression compared between non-injured (Day 0) and injured time points (Days 2, 5 and 7). Notably, *Npm1* and *Rpsa*, both frequently mutated in our data (Supplementary Table 1), were highly expressed in SCs at Days 2, 5 and/or 7 (Fig. 3e,f and Extended Data Fig. 3c,d). Previous studies showed *Npm1* degradation is required for early myogenesis in murine myoblasts^37^. *Rpsa*, which interacts with laminins, was previously identified as the top differentially expressed gene in SCs and as a key regulator of SC activation during aging^36^. *Gmpr*, mutated specifically in MSM mice, showed higher expression in mature muscle cells^36,40^, whereas *Pnkd*, also mutated in MSM mice, was enriched in SCs (Fig. 3f). Notably, missense *Pnkd* mutations are linked to movement disorders with prolonged muscle contractions^38,43–45^. We tried to match genes carrying somatic mutations with our previous work on somatic mutations in human aged SCs^12^, but no overlaps were found between the studies. A potential explanation for this was the limited amounts of cells analyzed in the previous study. In summary, our data reveal several SNVs that could impair muscle regeneration and function, though further studies are needed to confirm this.

## Reduced regeneration and strength in MSM and Msh2-def mice

**Fig. 4:**
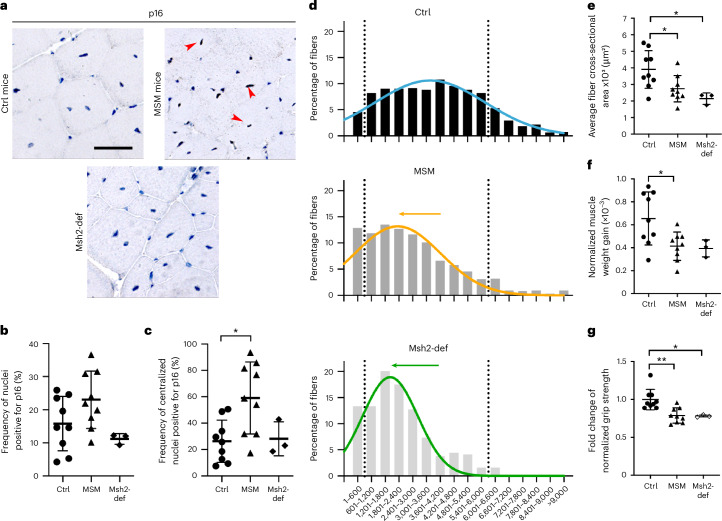
Impaired regeneration and reduced muscle function in MSM mice. **a**, p16 immunohistochemical staining of regenerated TA. Arrowheads indicate examples of p16-positive nuclei. **b**, Quantification of p16-positive cells in regenerated TA (Control *n* = 9, MSM *n* = 9, Msh2-def. *n* = 3); Control vs MSM *P* = 0.1616, Control vs Msh2-def. *P* = 0.6654. **c**, Quantification of p16-positive centralized nuclei in regenerated TA (Control *n* = 9, MSM *n* = 9, Msh2-def. *n* = 3); Control vs MSM *P* = 0.0122, Control vs Msh2-def. *P* = 0.9907. **d**, Graphs displaying the size distribution of regenerated fibers across the different groups. Curves illustrate the Gaussian distribution of fiber sizes. Arrows show the shift towards smaller fibers in MSM and *Msh2*-deficient mice (Control *n* = 9, MSM *n* = 9, Msh2-def. *n* = 3). **e**, Graph showing the average cross-sectional area of regenerated fibers (Control *n* = 9, MSM *n* = 9, Msh2-def. *n* = 3); Control *vs* MSM *P* = 0.04606, Control *vs* Msh2-def. *P* = 0.0277. **f**, Graph showing the difference in TA weight gain due to hypertrophy (Control *n* = 9, MSM *n* = 9, Msh2-def. *n* = 3); Control *vs* MSM *P* = 0.0244, Control *vs* Msh2-def. *P* = 0.0959. **g**, Graph showing the fold change of normalized grip strength (Control *n* = 9, MSM *n* = 9, Msh2-def. *n* = 3); Control *vs* MSM *P* = 0.0024, Control *vs* Msh2-def. *P* = 0.0250. Panel **a** scale bar indicates 100 μm; panel **d** scale bar indicates 20 μm. Statistical tests used in **b**, **c**, **e**, **f** and **g**: one-way ANOVA with Tukey multiple comparison test. * *P* < 0.05; ** *P* < 0.01; *** *P* < 0.001 Graphs in **b**, **c**, **e**, **f**, and **g** present data as mean ± s.d. Graph **d** presents data only as mean for clearer presentation. Source data

**Extended Data Fig. 4:**
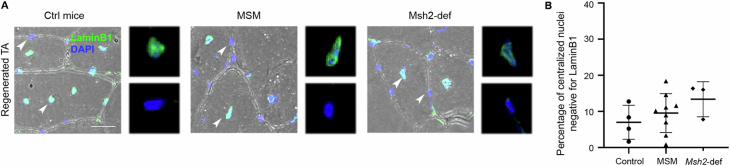
Measurement of muscle fiber atrophy. (**a**) Lamin B1 immunofluorescence of regenerated TA. Arrows indicate Lamin B1-positive and negative nuclei that are enlarged in the side pictures. Transmitted detection was used to visualize myogenic fibers. (**b**) Quantification of Lamin B1-positive centralized myonuclei in regenerated TA (Control n = 4, MSM n = 9, *Msh2*-def. n = 3); Control *vs* MSM P = 0.6948, Control *vs* *Msh2*-def. P = 0.2721. Panel **a** scale bar indicates 20 μm. Statistical test: **b**: one-way ANOVA with Tukey multiple comparison test. Graphs **b** presents data as mean ± SD. Source data

**Extended Data Fig. 5:**
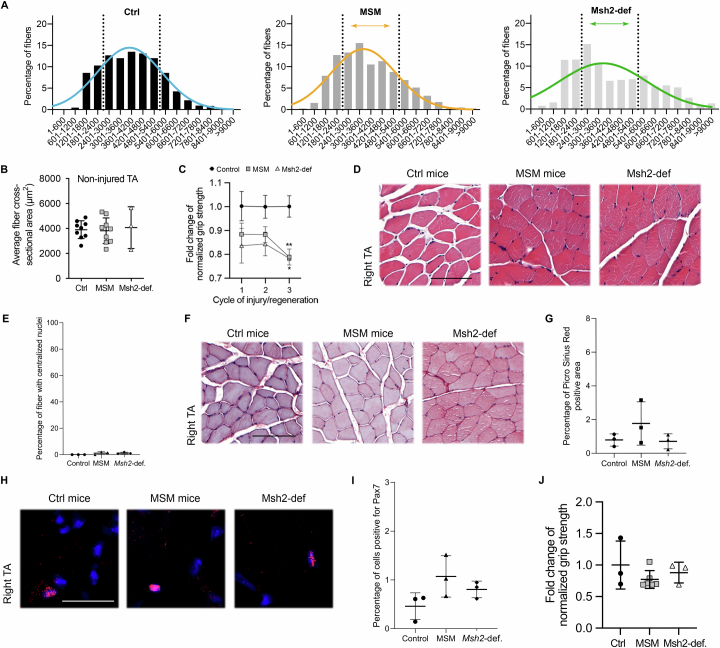
No abnormal myofiber sizes in non-injured TA from MSM and *Msh2*-deficient mice. (**a**) Graphs displaying the size distribution of fibers across the different groups after 3 regeneration cycles in the contralateral non-injured TA. Curves illustrate the Gaussian distribution of fiber sizes. Double arrows show no shift in fiber size in MSM and *Msh2*-deficient mice compared to controls (Control n = 9, MSM n = 9, *Msh2*-def. n = 3). (**b**) Graph showing average cross-sectional area of fibers after 3 regeneration cycles regeneration in the contralateral non-injured TA (Control n = 9, MSM n = 9, *Msh2*-def. n = 3); Control *vs* MSM P = 0.9844, Control vs *Msh2*-def. P = 9665. (**c**) Graph showing the fold change of normalized grip strength after every cycle of injury/regeneration in control, MSM and *Msh2*-deficient mice (Control n = 9, MSM n = 9, *Msh2*-def. n = 3); Control *vs* MSM P = 0.0048, Control *vs* *Msh2*-def. P = 0.0030. (**d**) Hematoxylin and eosin staining of non-injured right TA obtained from non-injured mice. (**e**) Quantification of centralized nuclei from non-injured right TA obtained from non-injured mice (Control n = 3, MSM n = 3, *Msh2*-def. n = 3); Control *vs* MSM P = 0.2104, Control *vs* *Msh2*-def. P = 0.1842. (**f**) Picro Sirius Red staining of non-injured right TA. (**g**) Quantification Picro Sirius Red-stained muscle connective tissue from non-injured right TA obtained from non-injured mice (Control n = 3, MSM n = 3, *Msh2*-def. n = 3); Control vs MSM P = 0.3673, Control vs *Msh2*-def. P = 0.9907. (**h**) Pax7 immunofluorescence of right non-injured TA obtained from non-injured mice. (**i**) Quantification of Pax7-positive cells in right non-injured TA obtained from non-injured mice (Control n = 3, MSM n = 3, *Msh2*-def. n = 3), Control *vs* MSM P = 0.1105; Control *vs* *Msh2*-def. P = 0.4106. (**j**) Graph showing the fold change of normalized grip strength in uninjured mice after 3 cycles of injury and regeneration in non-injured (Control n = 3, MSM n = 9, *Msh2*-def. n = 3); Control vs MSM P = 0.3580, Control vs *Msh2*-def. P = 0.7917. Statistical test: **b**, **e**, **g**, **i**, **j**: one-way ANOVA with Tukey multiple comparison test; C: two-way ANOVA with Tukey multiple comparison test. Graph **a** presents data only as mean without SD for clearer presentation. Graphs **b**, **c**, **e**, **g**, **i**, **j** present data as mean ± SD. Panels **d** and **f** scale bars indicate 100 μm; panel H scale bar indicates 20 μm. Source data

Previous work showed that increased mutation burden impairs SC proliferation and differentiation in vitro^12^. To assess functional effects in vivo, we analyzed muscle tissue from the cellular to the organ level. Given the role of senescence in tissue dysfunction^1,26^, we stained for p16 and found increased p16-positive cells in MSM mice, especially in centralized nuclei, whereas levels were unchanged in *Msh2*-deficient muscles (Fig. 4a–c). This suggests a DSB-linked senescence response in MSM mice not present in *Msh2*-deficient mice. However, Lamin B1 staining did not indicate a statistically significant increase in senescence induction in MSM or *Msh2*-deficient regenerated muscles (Extended Data Fig. 4a b). Regenerated muscle fibers remained smaller in MSM mice, whereas control mice achieved a normal size (Fig. 4d,e), suggesting defective muscle regeneration in MSM (Fig. 4d,e). A similar myofiber size reduction was observed in *Msh2*-deficient mice (Fig. 4d,e). Importantly, no size differences were seen in contralateral, non-injured muscles, indicating that the DNA repair deficiencies in SCs affected regeneration following injury regeneration and SC propagation (Extended Data Fig. 5a,b).

Our strategy to study increased somatic mutation accumulation focused on SCs of the TA muscle. However, we asked whether the regeneration defect extended to whole-muscle function. Hypertrophy is normally seen after injury regeneration^3^ and although there was muscle weight gain in the MSM mice it was lower than in the controls (Fig. 4f). Finally, lower-than-control grip strength in MSM mice throughout the experiment showed that muscle with increased somatic mutations had reduced grip strength (Fig. 4g). Grip strength was mostly reduced after the last cycle of regeneration compared to the previous two cycles, when the number of induced somatic mutations is expected to peak (Extended Data Fig. 5c). *Msh2*-deficient mice showed similarly reduced muscle mass and strength (Fig. 4f,g and Extended Data Fig. 5c), indicating functional impairment at the organ level.

To confirm that the observed phenotype depended on SC propagation to muscle fibers, we analyzed control, MSM and *Msh2*-deficient mice that did not undergo injury. Recombination rates were low (1.92% and 1.72%, respectively; Fig. 1d), indicating minimal regeneration. Thus, only SCs and possibly a few regenerated muscle fibers, at most, will harbor more somatic mutations. The right TA from these mice did not show statistically significant changes in the number of centralized nuclei (Extended Data Fig. 5d,e), collagen deposition (Extended Data Fig. 5f,g) and total number of SCs (Extended Data Fig. 5h,i) suggesting that growth or activity alone did not impair regeneration in the absence of injury. Grip strength in uninjured MSM and *Msh2*-deficient mice was similar to uninjured controls, indicating that widespread propagation of mutation-bearing SCs during regeneration is required to impair muscle regeneration (Extended Data Fig. 5j).

## Discussion

This study models excessive somatic mutation accumulation during postnatal skeletal muscle regeneration. Using two in vivo mouse models, we show that elevated genomic instability and somatic mutation burden impair muscle regeneration and reduce muscle strength.

Both MSM and *Msh2*-deficient mice exhibited similar phenotypes, including reduced muscle fiber cross-sectional area, lower hypertrophy and lower grip strength. However, MSM mice showed additional regeneration defects, notably increased collagen deposition in regenerated muscle. Although chronic FAP expansion can contribute to fibrosis^30,31^, Pdgfrα staining revealed no change in FAP numbers between uninjured and regenerated TA. Further studies are needed to determine the cause of the higher collagen deposition in MSM mice, such as altered FAP differentiation or disrupted signaling between myofibers, extracellular matrix, and FAPs.

Both models accumulated excessive SNVs, but only MSM mice accumulated large SVs. Interestingly, *Msh2*-deficient mice had a higher SNV load (2.77x versus 1.61x in MSM). One possible explanation for this could be that SVs in MSM mice lead to the loss of cells with excessive mutations. The lower SNV count also correlates with lower *Msh2* recombination efficiency in MSM mice (13.33%) compared to *Msh2*-deficient mice (21.65%).

MSM mice showed higher levels of DSB and single-stranded DNA, consistent with the *Blm* deletion inducing the occurrence of large SVs. *Msh2*-deficient mice showed impaired muscle regeneration and reduced muscle strength comparable to the MSM model, but with less pronounced DNA damage. Low levels of DNA damage were expected in the *Msh2*-deficient mice, as the function of the Msh2 protein is to recognize mismatched base-pairs during DNA replication^46^. Without Msh2, mismatches remain undetected and eventually become mutations. Although Msh2 also participates in additional repair pathways (that is, nucleotide excision repair and base excision repair)^47^, which could have been a source of additional DNA damage, staining of phosphorylated RPA (S4/S8) suggested there was no substantial DNA damage in *Msh2*-deficient mice. *Msh2*-deficient mice showed no increase in p16 or Lamin B1-related senescence in regenerated muscle, indicating senescence wasn’t a main factor in reduced regeneration and strength. Overall, these findings support that somatic SNVs accumulation can impact tissue dysfunction.

By integrating somatic mutation data from our mice and reanalyzing publicly available single-cell data on muscle regeneration^42^, we mapped 13 exonic SNVs to genes related to muscle biology. Although their allele frequencies were consistent with the myonuclei proportion in muscle tissue (13–32%), these somatic mutations could have potentially occurred in other cell types in the muscle. Particularly during muscle regeneration, FAPs and immune cells expand substantially^36,42^, and extensive DNA replication could generate more mutations independently of the *Blm* and *Msh2* knock-out. Mutations in muscle fibers likely arose from clonal SC expansion during regeneration. Mutations neutral to SC proliferation may impair subsequent SC differentiation or reduce muscle strength, whereas others could give SCs a growth advantage, disrupting the balance needed for effective regeneration. The 13 exonic SNVs affect muscle-related genes expressed during regeneration, suggesting a role in impaired repair. However, detecting the full spectrum of somatic mutations in a tissue is still challenging, particularly with bulk analysis, which detects clonally expanded mutations^2,48,49^. Hence, additional undetected deleterious mutations likely contributed to the phenotype.

Our study provides experimental evidence that somatic mutation accumulation in post-zygotic cells can contribute to muscle aging phenotypes. It highlights the role of somatic mutations in age-related diseases and a consequent impact of donor age on progenitor cell fitness for transplantation. This model could be applied to other cell types, to explore somatic mutation effects on tissue function and maintenance.

## Methods

### Ethical approval

Animal studies were approved by Linköping’s regional animal research ethical review board (Dnr. ID 215, Dnr. ID 04483-2023). All procedures were performed in accordance with the institutional guidelines and regulations.

### Experimental mice

All experimental mice from this study belonged to the C57BL/6 J strain. Mice were housed in a pathogen-free animal facility at the Karolinska Institutet (Campus Flemingsberg, Sweden) and maintained in a 12 h light/dark cycle, at 20–22 °C temperature and 50–65% air humidity. *Pax7*^CreERT2^ mice were purchased from The Jackson Laboratory and were published before^3^. *Msh2*^LoxP^ mice were a kind gift from W. Edelmann^4^. *Blm*^tm4Ches^ mice were a kind gift from A. J. R. Bishop^5^. All three transgenic lines were crossed to obtain the MSM mice that genotyped heterozygous for *Pax7*^CreERT2^, homozygous for *Msh2*^LoxP^ and homozygous for *Blm*^tm4Ches^ (*n* = 9; 5 females, 4 males). The *Msh2-*deficient group of animals was heterozygous for *Pax7*^CreERT2^ and homozygous for *Msh2*^LoxP^ (*n* = 3; 2 females, 1 male). The control mice group was heterozygous for *Pax7*^CreERT2^ and didn’t carry any *Msh2*^LoxP^ or *Blm*^tm4Ches^ alleles (*n* = 9; 5 females, 4 males).

Muscle injury and Cre activation were performed as described by Murphy et al.^3^. Briefly, muscle injury was induced by injecting 25 µl of 1.2 % BaCl_2_ (Sigma-Aldrich) in saline into the right TA muscle. The left TA served as the non-injured control. Three consecutive 28-day muscle injury and regeneration cycles were performed by inducing muscle injury on days 0, 28 and 56 (Fig. 1a). During each of the cycle each mouse received an intraperitoneal injection of 0.1 mg of tamoxifen (Sigma-Aldrich) per gram of body weight dissolved in corn oil (Sigma-Aldrich) on the 4th and 3rd day before the injury, on the same day as the injury, and on the two following days. The goal of the repeated tamoxifen injections was to maximize the number of SCs that were recombined. A weekly tamoxifen injection was also given for the following three weeks before the start of the next muscle injury and regeneration cycle. These number of tamoxifen injections were previously shown to induce recombination in over 95% of SCs^3^, a number that would maximize the number of recombined myofibers upon regeneration. The grip strength test was performed before the first and for 6 weeks after the end of the last regeneration cycle. We used an in-house developed grip strength meter based on a conventional 25 N force meter (Sauter) to measure the grip strength of hind two legs. A group of non-injured control (*n* = 3; 3 males), MSM (*n* = 6, 3 females, 3 males) and Msh2-deficient (*n* = 3; 3 males) mice underwent the same experimental procedure and were used as additional controls to assess skeletal muscle strength when somatic mutations were limited to SCs in a context of aging. All mice were aged 7-9 weeks at the start of the experiment. The average of the last 4 grip strength measurements was compared to the first measurement before the first injury. Body weight was used for normalization. The experiment lasted for 15 weeks, and mice were euthanized on day 100 by administering an overdose of isoflurane (Baxter). Right and left TA were carefully removed, weighed with an analytical balance, and halved; one part was flash-frozen in liquid nitrogen and the other fixed with 4 % paraformaldehyde (Merck).

Genotyping was performed using the following primers:

- Pax7^CreERT2^ mice: forward primer 5′-GCTGCTGTTGATTACCTGGC-3′, reverse primer 1 5′-CTGAGACAGGACCG-3′ and reverse primer 2 5′-CAAAAGACGGCAATATGGTG-3′.
- *Msh2*^LoxP^ mice: Msh2 forward 5′-TACTGATGCGGGTTGAAGG-3′, Msh2 reverse: 5′-AACCAGAGCCTCAACTAGC-3′.
- *Blm*^tm4Ches^ mice: Blm forward 5′-AACCTGCTTCAGCTAGGAGCTTCC-3′, Blm reverse 5′-TGGGACCGAATTGCTTCAACAACG-3′.

### Tissue processing and histology

Fixed TA samples were embedded in paraffin and cut into 4 μm sections. Hematoxylin and eosin staining was performed according to established procedures and used for fiber cross-sectional area analysis. At least 100 fibers per sample were quantified, using “Polygon selection” followed by “Analyze” and “Measure” commands in ImageJ. For regenerated samples, only areas with fibers presenting centralized nuclei were included in the measurements. Detection of collagen was performed by Picro Sirius Red staining. Briefly, sections were deparaffinized and incubated with 0.1% Picro Sirius Red stain for 20 min and then counterstained with Mayer’s hematoxylin. The percentage of collagen in the tissue was calculated by selecting the red-stained portion of each image using Adobe Photoshop, deleting all non-red pixels, converting the resulting image to Bitmap format and quantifying the selected pixels with ImageJ “Analyze particles” function.

### Immunostaining

Muscle sections (4 μm) were deparaffinized and subjected to heat-induced epitope retrieval. Blocking was performed with normal goat/donkey serum and/or mouse-to-mouse blocking reagent (Scytek). Primary antibodies were applied to specimens, followed by overnight incubation. Primary antibodies used were: rabbit anti-Msh2 (1:500, ab70270, Abcam), to validate the *Msh2* deletion, rabbit anti-Blm (1:100, ab2179, Abcam), to validate the *Blm* deletion, mouse anti-Pax 7 (1:100, DSHB) to identify SCs, mouse anti-Pcm1 (1:100, sc-398365, Santa Cruz Biotechnology) to detect nuclei specifically from myofibers, rat anti-PDGFRα (1:150, 14-1401-82, Thermo Fisher Scientific) to detect fibroadipogenic progenitors, rabbit anti-53BP1 (1:1,500, ab36823, Abcam) to detect DNA damage, rabbit anti RPA32/RPA2 (phospho S4 + S8, 1:100, ab87277, Abcam) to detect single-stranded DNA, rabbit anti-Ki67 (1:150, ab15580, Abcam), to detect proliferative cells, and rabbit anti-p16 (1:400, M-156, sc1207, Santa Cruz Biotechnology) and rabbit anti-LaminB1 (1:500, ab16048, Abcam), both to mark senescent cells. Sections were then incubated with the appropriate secondary antibodies: Alexa Fluor 568, donkey anti-rabbit (1:500, #A10042); Alexa Fluor 647, goat anti-mouse (1:500, #A21236); Alexa Fluor 647, goat anti-rabbit (1:500, #A21245); Alexa Fluor 488, goat anti-rat (1:500, #A11006). For immunohistochemistry, vectastain ABC kit (ABC Elite, Vector Laboratories) and DAB solution (Dako) were used for enzymatic detection. The secondary antibody was Biotin-goat anti rabbit (1:2,000, 656140, Invitrogen) Tissue sections were counterstained with either Mayer’s hematoxylin (Histolab) or DAPI (Thermo Fisher Scientific). For each staining, the percentage of positive nuclei per sample was obtained by manually scoring 200–600 DAPI-positive nuclei from three different images. Centralized and non-centralized nuclei were manually evaluated. Quantifications were done in a double-blind fashion.

### Image acquisition

Histological and immunohistochemistry imaging was achieved using the Nikon Eclipse E1000 microscope (Nikon Corporation) coupled to the Nikon digital sight DS-Fi2 camera. Immunofluorescence imaging was performed on a Nikon A1R and A1+ single point scanning confocal, using the NIS Elements software (Nikon Corporation), and imaging with a ×20 air objective. For each sample, we acquired large images of 2×2 tiles, which were stitched in one final image. We also captured Z-stacks over 8 planes, with 1.5 um separation between adjacent sections. Maximum intensity projection (MaxiIP) images were generated from the Z-stacks and analyzed. To detect centralized nuclei we used Transmitted Detection pictures to identify the fiber structure. Analysis of confocal images were performed using NIS elements Viewer, NIS Elements AR v5.42.02 and QuPath 0.5.1 (ref. ^50^). Immunofluorescence images were blinded.

### DNA isolation for recombination analysis and sequencing

We used low tissue input for the DNA extraction. Twelve 50 µm sections of formalin-fixed and paraffin-embedded regenerated TA samples were cut and DNA was extracted using Gentra Puregene Tissue Kit (Qiagen) according to manufacturer’s instructions for DNA purification from formalin-fixed and paraffin-embedded tissue. For use as reference for identification of somatic mutations, DNA was also extracted from frozen spleen samples of the respective mice using the same kit according to manufacturer’s instructions for DNA purification from tissue.

### Recombination analysis

Analysis of recombined alleles was performed using the QX200 droplet digital PCR system (Bio-Rad). PCR reactions were set up using 2x QX200 EvaGreen Mix (Bio-Rad), forward and reverse primers and template DNA and ran according to manufacturer’s instructions. 10 ng of sample DNA was used per well and run in two replicate wells. Primers were designed to be specific for the *Msh2* and *Blm* recombined alleles (*Msh2* forward: 5′-TACTGATGCGGGTTGAAGG-3′, reverse: 5′-GGCAAACTCCTCAAATCACG-3′, previously published^11^; *Blm* forward: 5′-TCAATGGCAAAAATAGGGAGGGTTG-3′, reverse: 5′-TGGGACCGAATTGCTTCAACAACG-3′). Recombined allele counts were normalized against *Egfr* allele (*Egfr* forward: 5′-CCACAGCTGAAAATGCAGAG-3′, reverse: 5′-CCTCACCATGAGGCAAACTT-3′). The data from ddPCR were analyzed using QuantaSoft v.1.6.

### Sequencing

WGS was performed on DNA extracted from skeletal muscle and spleen from injured control, MSM and Msh2-deficient mice (*n* = 3). Because somatic mutations remain challenging to detect^2,48^, we used bulk-tissue WGS to identify clonally expanded mutations, rather than measuring the somatic mutation burden of individual cells. Sequencing was performed by the National Genomics Infrastructure in Stockholm, Sweden, and the SNP&SEQ Technology Platform in Uppsala, Sweden. From the spleen, sequencing libraries were prepared from 1 μg DNA using the TruSeq PCR-free DNA sample preparation kit (Illumina Inc.) targeting an insert size of 350 bp. The library preparation was performed according to the manufacturers’ instructions. From the skeletal muscle, libraries were prepared from 50 ng of DNA using the SMARTer ThruPLEX DNA-seq Prep Kit (Takara Clontech) according to the manufacturers’ protocol. The libraries were sequenced on HiSeqX, paired-end 150 bp read length, v2.5 sequencing chemistry.

### Somatic mutation detection from downsampled data

Fastq files were aligned to the mouse reference genome (mm10, GRCm38) using BWA^51^ 0.7.17-r1188 with default parameters. Alignments were sorted and indexed using SAMtools 0.1.19 (ref. ^52^). The average coverage was 42X. Analysis of the sequencing data revealed differences in coverage between the samples. Downsampling was necessary to be able to compare the proportion of detectable somatic mutations between the different samples due to a difference in coverage between the samples. BAM files were therefore randomly downsampled to the lowest coverage using SAMtools (46X for muscle, 29X for the spleen). For every sample, duplicated reads were marked using Picard (https://broadinstitute.github.io/picard/, v2.10.3-SNAPSHOT). Local realignment around InDels was performed and the two samples from the same individual mouse were aligned together to improve variant calling using the Genome Analyzer Toolkit v3.4.0 (GATK)^53^. Variants were called using the somatic mode of three somatic callers: GATK-MuTect2 (v.4.1.1.0)^54^, Strelka2 (v.2.9.3)^55^ and Varscan2 (v.2.3.7)^56^ with skeletal muscle as tumor and spleen as normal. Annotations were done using snpEff_4.2 (ref. ^57^) and VEP Ensembl^58^. Somatic variants detected by all three somatic variant callers were subjected to post-process filtering. Variants that had minimum 15X read depth in spleen and skeletal muscle, minimum five alternative allele depth in the skeletal muscle, and a statistically significant difference between the spleen read count and the skeletal muscle read count (*P* < 0.05, Fisher exact test) were considered somatic variants. All recurrent variants were excluded from the analysis. For SV detection, DELLY2 was used with default parameters^59^. We also excluded SNVs from lab strains using dbSNP142 from the mouse genome project.

### Somatic mutation detection from non-downsampled data

To identify somatic variants, the above analysis and filtering were also performed on non-downsampled data, so the amount of information for each individual population (control, *Msh2*-deficient, and MSM mice) would be maximized. Using Varscan2, we selected somatic variants that had at least 10X coverage in spleen samples, 5X coverage in skeletal muscle samples for the alternative allele and a *P* < 0.05. Additionally, variants from Varscan2 were selected only if also detected by GATK-MuTect2 and Strelka2 variant callers. We also excluded SNVs from lab strains using dbSNP142 from the mouse genome project. Using snpEFF, the obtained variants were annotated to find SNV-associated genes. MutPred2 (ref. ^35^) was used to predict the pathogenicity of missense variants. For SV detection, DELLY2 was used with default parameters^59^.

### Single-cell transcriptome analysis of skeletal muscle regeneration

The single-cell transcriptome (scRNA-seq) data from Chromium 10X generated by De Micheli et al.^42^ was reanalyzed by maintaining the same parameters specified in the published study. We used Seurat 3.2.0 (ref. ^60^) and performed shared nearest neighbor clustering with 0.4 resolution to reproduce the optimal clusters. We used uniform manifold approximation and projection to visualize the individual clusters and the gene expression profiles. The markers representing individual clusters were predicted using the “FindAllMarkers” function from the Seurat package. The expression of SNV-associated genes was verified in the scRNA-seq dataset.

### Statistics and reproducibility

Statistical analyses of the experimental results from the mice were performed using the following parametric tests: unpaired *t*-test, one-way and two-way ANOVA with Tukey or Bonferroni´s multiple comparisons test. The data distribution was assumed to be normal, but this was not formally tested. No statistical method was used to predetermine sample size. We used similar sample sizes to those reported in previous publications. These sample sizes were sufficient to test our hypothesis and perform statistical analysis. In addition, obtaining the different genotypes was challenging due to the use of triple transgenics and the attempt to have equal gender distributions within the sample groups. In addition, we always aim to follow the 3 Rs for animal research (refine, replace and reduce). The experiments were not randomized. Data collection was not performed blind to the conditions of the experiment. Blinding was used for immunofluorescence analysis. No animals or data points were excluded from the analysis. For the single-cell RNA-sequencing data, the non-parametric Wilcoxon test was used, using the Benjamini Hochberg method for adjusting *P* values for multiple testing. Experimental replicates are biological. Graphs were plotted as mean ± standard deviation using GraphPad Prism software version 8.0.2.

### Reporting summary

Further information on research design is available in the Nature Portfolio Reporting Summary linked to this article.

## Supplementary information

Reporting Summary

Supplementary Data 1 VCF files from both the downsampled and non-downsampled analysis containing all structural variation data.

Supplementary Data 2 VCF files from the non-downsampled analysis containing all detected SNVs.

Supplementary Table 1 Table containing all exonic SNV and additional information, such as type of mutation, allele frequency or MutPred2 score.

## Source data

Source Data Fig. 1 Raw data and statistical source data.

Source Data Fig. 2 Raw data and statistical source data.

Source Data Fig. 3 Raw source data.

Source Data Fig. 4 Raw data and statistical source data.

Source Data Extended Data Fig. 1 Raw data and statistical source data.

Source Data Extended Data Fig. 2 Raw data and statistical source data.

Source Data Extended Data Fig. 3 Raw data and statistical source data.

Source Data Extended Data Fig. 4 Raw data and statistical source data.

Source Data Extended Data Fig. 5 Raw data and statistical source data.

## Data Availability

The raw data have been deposited to NCBI and can be accessed using BioProject ID PRJNA793847 from https://www.ncbi.nlm.nih.gov/bioproject”. The data can be accessed directly via: https://www.ncbi.nlm.nih.gov/bioproject/PRJNA793847 The single-cell data derive from a previous study^39^ and were previously deposited in GEO with the accession number GSE143437. Mouse reference genome assembly GRCm38 (mm10) was used, obtained from the Genome Reference Consortium (GRC) release December 2011. dbSNP142 from the mouse genome project was used for SNP removal. All data supporting the findings of this study are available within the manuscript or are available from the corresponding authors upon request.
