# Supplementary material for: Induced somatic mutation accumulation during skeletal muscle regeneration reduces muscle strength
Source: Nat Aging. 2025 Aug 20;5(9):1739–49. doi: 10.1038/s43587-025-00941-y (PMC12443595; doi:10.1038/s43587-025-00941-y)
Supplement: Supplementary file 1 — Reporting Summary [file 43587_2025_941_MOESM1_ESM.pdf]

nature portfolio

Corresponding author(s): Maria Eriksson
Gwladys Revêchon

Last updated by author(s): June 17th, 2025

Reporting Summary

Nature Portfolio wishes to improve the reproducibility of the work that we publish. This form provides structure for consistency and transparency in reporting. For further information on Nature Portfolio policies, see our Editorial Policies and the Editorial Policy Checklist.

Statistics

For all statistical analyses, confirm that the following items are present in the figure legend, table legend, main text, or Methods section.

n/a	Confirmed	
☐	☒	The exact sample size (*n*) for each experimental group/condition, given as a discrete number and unit of measurement
☒	☐	A statement on whether measurements were taken from distinct samples or whether the same sample was measured repeatedly
☐	☒	The statistical test(s) used AND whether they are one- or two-sided<br>*Only common tests should be described solely by name; describe more complex techniques in the Methods section.*
☒	☐	A description of all covariates tested
☐	☒	A description of any assumptions or corrections, such as tests of normality and adjustment for multiple comparisons
☐	☒	A full description of the statistical parameters including central tendency (e.g. means) or other basic estimates (e.g. regression coefficient) AND variation (e.g. standard deviation) or associated estimates of uncertainty (e.g. confidence intervals)
☐	☒	For null hypothesis testing, the test statistic (e.g. *F*, *t*, *r*) with confidence intervals, effect sizes, degrees of freedom and *P* value noted<br>*Give P values as exact values whenever suitable.*
☒	☐	For Bayesian analysis, information on the choice of priors and Markov chain Monte Carlo settings
☒	☐	For hierarchical and complex designs, identification of the appropriate level for tests and full reporting of outcomes
☒	☐	Estimates of effect sizes (e.g. Cohen's *d*, Pearson's *r*), indicating how they were calculated

Our web collection on statistics for biologists contains articles on many of the points above.

Software and code

Policy information about availability of computer code

Data collection	Immunofluorescence images were collected using NIS Elements software (Nikon corporation, v5.42.02). Data was collected in Excel version 16.98
Data analysis	Analysis of confocal images were performed using NIS elements Viewer, NIS Elements AR v5.42.02 and QuPath 0.5.1<br><br>ddPCR data was analyzed using QuantaSoft V.1.6<br><br>Whole genome sequencing data: BWA 0.7.17 with default parameters (alignment to the mouse reference genome mm10, GRCm38), SAMtools 0.1.19 (sorting, indexing and downsampling), Picard MarkDuplicates (v2.10.3-SNAPSHOT), GATK v3.4.0 (realignment around InDels), GATK-MuTect2 v. 4.1.1.0, Strelka2 v.2.9.3, Varscan2 v.2.3.7 (somatic mutation caller); snpEff_4.2 and VEP Ensembl v99.2 (annotation), dbSNP142 (removal of SNVs from lab strains); MutPred2 (pathogenicity of SNV, DELLY2 with default parameters (detection of structural variation)<br><br>Single-cell RNA-sequencing: Seurat 3.2.0 was used for all analysis.

For manuscripts utilizing custom algorithms or software that are central to the research but not yet described in published literature, software must be made available to editors and reviewers. We strongly encourage code deposition in a community repository (e.g. GitHub). See the Nature Portfolio guidelines for submitting code & software for further information.

Data

Policy information about availability of data

All manuscripts must include a data availability statement. This statement should provide the following information, where applicable:

- Accession codes, unique identifiers, or web links for publicly available datasets
- A description of any restrictions on data availability
- For clinical datasets or third party data, please ensure that the statement adheres to our policy

Data availability
The raw data has been deposited to NCBI and can be accessed using BioProject ID PRJNA793847 from https://www.ncbi.nlm.nih.gov/bioproject”. The single-cell data derives from a previous study39 and was previously deposited in GEO with the accession number GSE143437. Mouse reference genome assembly GRCm38 (mm10) was used, obtained from the Genome Reference Consortium (GRC) release December 2011. dbSNP142 from the mouse genome project was used for SNP removal. The authors declare that all data supporting the findings of this study are available within the manuscript or are available from the corresponding authors upon request.

The sequencing data has been deposited, but it might take up to 2 weeks from June 16th 2025 for to be released. The data can be consulted using this link: https://www.ncbi.nlm.nih.gov/bioproject/PRJNA793847

Research involving human participants, their data, or biological material

Policy information about studies with human participants or human data. See also policy information about sex, gender (identity/presentation), and sexual orientation and race, ethnicity and racism.

Reporting on sex and gender	N/A
Reporting on race, ethnicity, or other socially relevant groupings	N/A
Population characteristics	N/A
Recruitment	N/A
Ethics oversight	N/A

Note that full information on the approval of the study protocol must also be provided in the manuscript.

Field-specific reporting

Please select the one below that is the best fit for your research. If you are not sure, read the appropriate sections before making your selection.

☒ Life sciences ☐ Behavioural & social sciences ☐ Ecological, evolutionary & environmental sciences

For a reference copy of the document with all sections, see nature.com/documents/nr-reporting-summary-flat.pdf

Life sciences study design

All studies must disclose on these points even when the disclosure is negative.

Sample size	"No statistical methods were used to pre-determine sample sizes but our sample sizes are similar to those reported in previous publications (Sagelius et al., J Med Genet. 2008, 45:794-801; Whisenant et al., Nat Commun. 2022 Jun 2;13(1):3068). Hence the sample sizes were sufficient to test our hypothesis and perform statistical analysis. Furthermore, the acquisition of the different genotypes was challenging given that we worked with triple transgenics and needed to have equal gender distributions within the different sample groups. In addition, our research follow the 3R for animal research (Refine, Replace and Reduce)."
Data exclusions	No data was excluded
Replication	Our measurements represent biological replicates, since several animals were included in each experimental group (injured mice: Control n = 9, MSM n = 9, Msh2-def n = 3, uninjured mice: Control n = 3; MSM n= 3, Msh2-def n = 3).<br>For histological analysis of the muscle, at least 100 fibers per mouse were quantified. For immunostaining, at least 200 nuclei were counted from each mouse. Grip strength measurements were performed at least 3 times in each occasion. All replication attempts were successful.
Randomization	Randomization was not required since all animals underwent the same experimental procedure
Blinding	No blinding was performed in the animal experiments due to the use of triple transgenic mice. The genotypes of interested were challenging to achieve, so mice were used upon availability. In addition, the researchers genotyping the mice are the same ones performing the experiments, making blinding difficult. Immunofluorescence image quantification was done in blind.

Reporting for specific materials, systems and methods

We require information from authors about some types of materials, experimental systems and methods used in many studies. Here, indicate whether each material, system or method listed is relevant to your study. If you are not sure if a list item applies to your research, read the appropriate section before selecting a response.

Materials & experimental systems

n/a	Involved in the study
☐	☒ Antibodies
☒	☐ Eukaryotic cell lines
☒	☐ Palaeontology and archaeology
☐	☒ Animals and other organisms
☒	☐ Clinical data
☒	☐ Dual use research of concern
☒	☐ Plants

Methods

n/a	Involved in the study
☒	☐ ChIP-seq
☒	☐ Flow cytometry
☒	☐ MRI-based neuroimaging

Antibodies

Antibodies used

Primary anibodies: rabbit anti-Msh2 (1:500, ab70270, Abcam), rabbit anti-Blm (1:100, ab2179, Abcam), mouse anti-Pax 7 (1:100, DSHB) , mouse anti-Pcm1 (1:100, sc-398365, Santa Cruz Biotechnology), rabbit anti-53BP1 (1:1500, ab36823, Abcam), rabbit anti RPA32/RPA2 (phospho S4 + S8, 1:100 , ab87277, Abcam) , rabbit anti-Ki67 (1:150 ab15880, Abcam), rabbit anti-p16 (1:400, M-156, sc1207, Santa Cruz Biotechnology) and rabbit anti-LaminB1 (1:500, ab16048, Abcam), and rat anti-PDGFRalpha (1:150, 14-1401-82, Thermofisher Scientific)

Secondary antibodies: Alexa Fluor 568, donkey anti-rabbit (1:500, #A10042); Alexa Fluor 647, goat anti-mouse (1:500, #A21236); Alexa Fluor 647, goat anti-rabbit (1:500, #A21245); Alexa Fluor 488, goat anti-rat (1:500, #A11006), Biotin-goat anti rabbit (1:2000, 656140, Invitrogen)

Validation

rabbit anti-Msh2 (ab70270, Abcam). Cited by: Ghosh, S. et al. Nonhematopoietic Nrf2 dominantly impedes adult progression of sickle cell anemia in mice. JCI Insight 1, e81090, 81090 (2016).

rabbit anti-Blm (ab2179, Abcam). Cited by: Petsalaki, E., Dandoulaki, M., Morrice, N. & Zachos, G. Chk1 protects against chromatin bridges by constitutively phosphorylating BLM serine 502 to inhibit BLM degradation. J. Cell Sci. 127, 3902–3908 (2014).

mouse anti-Pax 7 (DSHB). Cited by: Le Moal, E. et al. Macrophage-derived superoxide production and antioxidant response following skeletal muscle injury. Free Radic. Biol. Med. 120, 33–40 (2018).

mouse anti-Pcm1 (sc-398365, Santa Cruz Biotechnology). Cited by: Holdgaard, S. G. et al. Selective autophagy maintains centrosome integrity and accurate mitosis by turnover of centriolar satellites. Nat. Commun. 10, 4176 (2019).

rabbit anti-53BP1 (ab36823, Abcam). Cited by: Luebben, S. W., Kawabata, T., Johnson, C. S., O'Sullivan, M. G. & Shima, N. A concomitant loss of dormant origins and FANCC exacerbates genome instability by impairing DNA replication fork progression. Nucleic Acids Res. 42, 5605–5615 (2014).

rabbit anti RPA32/RPA2 (phospho S4 + S8) (ab87277, Abcam). Cited by: Herrtwich, L. et al. DNA Damage Signaling Instructs Polyploid Macrophage Fate in Granulomas. Cell 167, 1264-1280.e18 (2016).

rabbit anti-Ki67 (ab15580, Abcam). Cited by: Bhanja, P., Norris, A., Gupta-Saraf, P., Hoover, A. & Saha, S. BCN057 induces intestinal stem cell repair and mitigates radiation-induced intestinal injury. Stem Cell Res. Ther. 9, 26 (2018).

rabbit anti-p16 (1:400, M-156, sc1207, Santa Cruz Biotechnology). Cited by: Toribio, R. E. et al. The midregion, nuclear localization sequence, and C terminus of PTHrP regulate skeletal development, hematopoiesis, and survival in mice. FASEB J. Off. Publ. Fed. Am. Soc. Exp. Biol. 24, 1947–1957 (2010).

rabbit anti-LaminB1 (ab16048, Abcam). Cited by: Jang, S. W. et al. Casein kinase 2 is a critical determinant of the balance of Th17 and Treg cell differentiation. Exp. Mol. Med. 49, e375 (2017)

rat anti-PDGFRalpha (14-1401-82, Thermofisher Scientific). Cited by: Narvaez Del Pilar, O., Gacha Garay, M. J. & Chen, J. Three-axis classification of mouse lung mesenchymal cells reveals two populations of myofibroblasts. Dev. Camb. Engl. 149, dev200081 (2022).

Animals and other research organisms

Policy information about studies involving animals; ARRIVE guidelines recommended for reporting animal research, and Sex and Gender in Research

Laboratory animals	Experiments have been conducted on mice (Mus musculus, C57Bl6J). Pax7-CreERT2 mice were purchased from the Jackson Laboratory, Msh2-LoxP mice were obtained from Winfried Edelmann and Blm-tm4Ches mice were obtained from Alexander JR Bishop. The experiments were started when the mice were 7-9 weeks old.
Wild animals	No wild animals were used
Reporting on sex	Both males and females were included equally following the principles of the 3Rs (Refine, Replace, Reduce). No mice were excluded based on gender. No transgenes were included in the Y chromosomes to avoid gender biases within the populations.
Field-collected samples	The study did not involve field-collected samples
Ethics oversight	The animal studies are approved by Linköping's regional animal research ethical review board. All experiments follow the descriptions of our ethical permits (Dnr. ID 215, DNR. ID 04483-2023). Institutional guidelines and regulations were followed in every experiment. Our pathogen-free animal facility in located at the Campus Flemingsberg from Karolinska Institutet, Sweden. Mice are kept 20-22°C with 50-65% humidity, and they are subjected to a 12-hour light/dark cycle. The mice have continuous and free access to food and water. Daily monitoring is performed to ensure animal wellness

Note that full information on the approval of the study protocol must also be provided in the manuscript.

Plants

Seed stocks	*Report on the source of all seed stocks or other plant material used. If applicable, state the seed stock centre and catalogue number. If plant specimens were collected from the field, describe the collection location, date and sampling procedures.*
Novel plant genotypes	*Describe the methods by which all novel plant genotypes were produced. This includes those generated by transgenic approaches, gene editing, chemical/radiation-based mutagenesis and hybridization. For transgenic lines, describe the transformation method, the number of independent lines analyzed and the generation upon which experiments were performed. For gene-edited lines, describe the editor used, the endogenous sequence targeted for editing, the targeting guide RNA sequence (if applicable) and how the editor was applied.*
Authentication	*Describe any authentication procedures for each seed stock used or novel genotype generated. Describe any experiments used to assess the effect of a mutation and, where applicable, how potential secondary effects (e.g. second site T-DNA insertions, mosiacism, off-target gene editing) were examined.*
